# Generation and Characterization of Indoor Fungal Aerosols for Inhalation Studies

**DOI:** 10.1128/AEM.04063-15

**Published:** 2016-04-04

**Authors:** Anne Mette Madsen, Søren T. Larsen, Ismo K. Koponen, Kirsten I. Kling, Afnan Barooni, Dorina Gabriela Karottki, Kira Tendal, Peder Wolkoff

**Affiliations:** a The National Research Centre for the Working Environment, Copenhagen, Denmark; b Section of Environmental Health, Department of Public Health, Faculty of Health and Medical Sciences, University of Copenhagen, Copenhagen, Denmark; HKI and University of Jena

## Abstract

In the indoor environment, people are exposed to several fungal species. Evident dampness is associated with increased respiratory symptoms. To examine the immune responses associated with fungal exposure, mice are often exposed to a single species grown on an agar medium. The aim of this study was to develop an inhalation exposure system to be able to examine responses in mice exposed to mixed fungal species aerosolized from fungus-infested building materials. Indoor airborne fungi were sampled and cultivated on gypsum boards. Aerosols were characterized and compared with aerosols in homes. Aerosols containing 10^7^ CFU of fungi/m^3^ air were generated repeatedly from fungus-infested gypsum boards in a mouse exposure chamber. Aerosols contained Aspergillus nidulans, Aspergillus niger, Aspergillus ustus, Aspergillus versicolor, Chaetomium globosum, Cladosporium herbarum, Penicillium brevicompactum, Penicillium camemberti, Penicillium chrysogenum, Penicillium commune, Penicillium glabrum, Penicillium olsonii, Penicillium rugulosum, Stachybotrys chartarum, and Wallemia sebi. They were all among the most abundant airborne species identified in 28 homes. Nine species from gypsum boards and 11 species in the homes are associated with water damage. Most fungi were present as single spores, but chains and clusters of different species and fragments were also present. The variation in exposure level during the 60 min of aerosol generation was similar to the variation measured in homes. Through aerosolization of fungi from the indoor environment, cultured on gypsum boards, it was possible to generate realistic aerosols in terms of species composition, concentration, and particle sizes. The inhalation-exposure system can be used to study responses to indoor fungi associated with water damage and the importance of fungal species composition.

## INTRODUCTION

A review paper concluded that evident dampness or mold in buildings has been associated with multiple allergic and respiratory health effects; however, measured microbial agents in dust have limited suggestive associations (1). This might be due partly to the multitude of existing microbial species and to the fact that other changes occur concurrently with water damage development. Concentrations of airborne fungi from around 50 to 300 CFU/m^3^ air (2, 3) to 10^3^ to 10^6^ CFU/m^3^ air (4–6) have been reported in the indoor environment. For exposure of the airways to occur, fungal particles must be aerosolized from infested surfaces. Airflows (7–9) and vibrations of building constructions (10) are expected to mediate fungal spore aerosolization. Indoor activities are expected to mediate spore resuspension from surfaces.

Mice are often used as model organisms to examine the immune mechanisms associated with fungal clearance, infection, tolerance, and allergy (11). This has the advantage, compared to epidemiological studies, that many factors can be controlled. In studies with mice as model animals, fungal exposures between 10^4^ and 10^8^ spores per mouse have been used (Table 1). Within the last few years, epidemiological (12–14) and case (15) studies have focused on the importance of fungal diversity, fungal composition, or indicator fungi on health effects. In studies with mice, the focus has been limited to very few fungal species and to studies involving exposure to one species at a time (Table 1). Similarly, studies of aerosolization of fungal particles have focused on one species at a time (8, 16).

**TABLE 1:** *In vitro* studies with mice as the model animals exposed to fungi or fungal toxins^*a*^

Fungus	Growth matrix^*a*^	Exposure method	Exposure concn	Studied effect	In relation to	Reference
Alternaria alternata or Aspergillus versicolor	NM	Intranasally	Culture extracts	Inflammation mechanisms	Asthma in industrialized countries	71
Alternaria alternata or Aspergillus versicolor	Agar: CMA and PDA	Nasal cavity	20 μl with 10^7^ spores/ml/mouse	Inflammation	Inhalation of fungi and rhinosinusitis	72
Aspergillus fumigatus	Agar: SDA	Intratracheally	10^7^ heat-killed or live conidia/mouse	Inflammation	Inhalation of fungi	73
A. fumigatus	Agar: SDA	Intranasal inoculation	10^7^ washed conidia/mouse	Inflammation	Allergic airway disease	74
A. fumigatus	Agar: SDA	Intratracheal injection	2 × 10^8^ washed or swollen conidia or 10^5^ hyphae/mouse	Transport and infection	Inhalation and transplant recipients	75
A. fumigatus	NM	Intratracheal	5 × 10^6^ conidia/mouse	Inflammation and allergy	Indoor and outdoor exposure	76
A. fumigatus	Agar: SDA	Intranasally	5 × 10^6^ conidia/mouse	Inflammation	Fungal exposure and asthma	77
A. fumigatus	Agar: SDA	Intranasally	2 × 10^6^ conidia/mouse	Inflammation and clearance	Inhalation and inflammation	78
A. fumigatus	Agar: SDA	Inhalation	3 × 10 min dead or alive conidia	Asthma	Indoor and outdoor exposure	27
A. fumigatus or A. versicolor	Agar: MEA	Involuntary Aspiration	Living or dead conidia, 2 × 10^6^/mouse	Inflammation	Indoor and outdoor exposure	26
A. versicolor	Agar: MEA	Intratracheal instillations	10^5^–10^8^ spores/mouse	Inflammation	Exposure in moisture damaged buildings	79
Cladosporium cladosporioides or A. versicolor	Agar: MEA	Intratracheally	10^4^ or 10^6^ spores/mouse, 3 times	Inflammation	Exposure in the homes	80
Fusarium oxysporum	Agar: SDA	Intravenous	10^7^ spores/mouse	Infection	Immunocompromised patients	81
Penicillium brevicompactum or P. chrysogenum	NM	Intratracheal	Toxins and metabolites	Inflammatory and cytotoxic responses	Exposure in damp buildings	82
P. chrysogenum	Nylon filter on agar: PDA	Deposited in oropharynx, mice inhaled the extract	Different weights of fungal antigen extract	Inflammation	Dampness in buildings	83
Stachybotrys atra	Agar	Intranasal injection	Toxins or 10^6^ spores	Inflammation	Damp houses	84
S. chartarum	NM	Intratracheal instillations	7 × 10^4^ spores/mouse	Changes in alveolar cells	Building occupants	85
S. chartarum	Broth: cellulose	Aerosols from extracts in water	Extracts from spores	Irritation in the airways	Fungal growth in buildings	86

a Abbreviations: CMA, corn meal agar; SDA, Sabouraud dextrose agar; MEA, malt extract agar; PDA, potato dextrose agar; NM, not mentioned.

Building constructions are expected to be exposed to several species simultaneously, and studies have revealed several fungal genera on indoor surfaces (17) and in settled dust (18); less is known at the species level and for airborne fungi. Different numbers of fungal spores can be aerosolized per fungus-infested area from different species (10, 19), and the relative humidity (RH) affects the number of spores aerosolized as a response to exposure to an airflow (7, 8, 20).

Fungi can adapt to growth in different environments, and factors of importance for virulence are affected by growth conditions; examples are melanin content (21, 22), mycotoxin (23, 24), and extracellular enzyme (25) production. The germinability (26) and viability (27) of conidia of Aspergillus fumigatus affect the response or persistence of conidia in lungs of mice. In studies of the health effects of fungal exposure, using mice as model organisms, fungi are typically grown on agar media and in cultures of only one fungal species, and fungal spores are often washed before exposure (Table 1). The exposure of spores to water may remove extracellular enzymes or, as seen for some species, induce conidial germination (28). Even though inhalation of fungal particles is considered the main exposure route, intratracheal or nasal instillation is often applied (Table 1).

Airborne fungal spores typically have an aerodynamic diameter (d_ae_) of 2 to 4 μm (29), and some species release fragments of 0.3 to 1.3 μm (8, 30, 31). Airborne Penicillium and Cladosporium spores have a diameter of 2 μm and 3 μm, respectively (32). Experimental data for mice show that about 61.7%, 4.5%, and 0.2% of particles of 5.0 μm deposit in the oral/nasal, tracheal/bronchial, and lung region, respectively. The same fractions for particles of 3.0 μm are 46.6%, 4.7%, and 1.1%, and for particles of 1.0 μm they are 58.0%, 8.2%, and 5.9% (33). For humans, about 87%, 85%, and 30% of particles with d_ae_ of 5 μm belong to the inhalable, thoracic, and respirable fractions. The same fractions for particles of 3.0 μm are 92%, 92%, and 74% (34). Furthermore, about 87% of particles with a d_ae_ of 5 μm deposit in the oral/nasal region, while about 78% of particles of 3.0 μm deposit in the oral/nasal region (34).

The aim of this study was to develop an inhalation exposure system allowing responses to exposure of the airways to mixed airborne indoor fungi to be examined in a head-only mouse exposure system. The system requirements were as follows: (i) fungi should be aerosolized *in situ* from growth on a building material, (ii) the fungal species composition should reflect species found in Danish homes, (iii) the variation in exposure concentration should simulate the variation that a person could experience within 1 h in an indoor environment, (iv) the exposure should last for 1 h, (v) it should be possible to alter the water content of the building material, (vi) it should be possible to take out subsamples from the aerosol chamber to identify airborne fungi, and finally (vii) it should be possible to measure the particle concentration in a time-dependent manner. As references, we studied the variation in fungal exposure in a home environment in terms of concentration and species, and furthermore, we identified the dominating airborne fungal species in airborne dust samples from 27 homes.

## MATERIALS AND METHODS

An outline of the study is presented in Table 2. The study is divided into three parts.

**TABLE 2:** Outline of the study^*a*^

Characteristic	Part I	Part II	Part III
Study target or activity	Fungal species in Danish homes. Variation in exposure to fungi in homes in 7-min intervals.	Aerosolization of fungal particles from gypsum boards colonized by fungi as a function of time and RH	Generation of fungal aerosols in an exposure chamber and characterization of generated aerosols
Level	27 homes + 2 homes	Gypsum boards, LAF bench	Gypsum boards, exposure chamber
Method(s)	Sampling for species: EDC; sampling for variation and species: DGI. Quantification: cultivation. Identification: MALDI-TOF.	Sampling: EDCs in homes and offices.Inoculation: dust suspension on gypsum boards. Humidity: 50% of the cultures were dried out by lowering the RH. Generation of aerosols: P-FLEC with two airflows. Particle measurement: APS. Sampling: GSP samplers. Quantification: cultivation.	Sampling for inoculation: EDCs in a home and offices. Inoculation: dust suspension on gypsum boards. Generation of aerosols: P-FLEC with two airflows. Particle measurement: APS. Sampling: ELPI, GSP, MINI. Quantification: cultivation and LAL assay. Identification: MALDI-TOF. Microscopy: ESEM.
Size and scope	27 homes (species); 2 homes during and between 4 activities (variation and species)	Gypsum boards infested with fungi in dust from 2 offices (sample A, 23 boards), 2 homes (sample B, 60 boards and sample C, 12 boards). Two RHs.	Gypsum boards infested with fungi in dust from 2 offices (sample A, 48 boards), 2 homes (sample C, 48 boards and sample D, 60 boards). Two RHs.

a Abbreviations: DGI, Dekati gravimetric impactor, a high-volume sampler; MALDI-TOF, matrix-assisted laser desorption ionization–time of flight; RH, relative humidity; LAF, laminar flow cabinet; P-FLEC, particle field and laboratory emission cell; APS, aerodynamic particle sizer; GSP, Gesamtstaub-probenahme, an aerosol sampler; EDC, electrostatic dust collector; ELPI, electrical low-pressure impactor; MINI, micro-inertial impactor; ESEM, environmental scanning electron microscopy.

### Exposure to fungal species in homes (part I).

As a background for the exposure model, knowledge on fungal species in airborne settled dust in Danish homes was determined. Fungi were identified in dust sampled using electrostatic dust cloths (EDCs; Zeeman Alphen, Netherlands); fungi were cultivated, and 316 isolates were identified using matrix-assisted laser desorption ionization–time of flight (MALDI-TOF) as described below. The dust was sampled in the living rooms in 27 dwellings in the greater Copenhagen area from December (early winter) to May (late spring) with a sampling time of 27 to 28 days; the content of endotoxin and CFU of fungi as well as the homes are described elsewhere (35).

### Variation in exposure level in homes (part I).

The aim of the study was to obtain knowledge about the variation in exposure to fungi during normal indoor activities as a background for variation in concentration and species (examined in part III). To measure short-term variation in exposure, we used the Dekati gravimetric impactor (DGI), a high-volume sampler (Dekati, Finland). The sampler classifies particles into four size fractions with lower cutoff points of aerodynamic diameters (d_ae_) of 0.264, 0.608, 1.200, and 2.968 μm when used at a flow rate of 50 liters/min. The particles in the two smallest-size fractions were not used. Airborne particles were sampled on polycarbonate filters (47-mm diameter; pore size, 1 μm; Nuclepore; Whatman) in November 2013. Sampling was performed in 7-min sampling periods before and during the following activities in one or two homes (abbreviated home I and home II): bed making, including shaking of the duvets; cleaning the kitchen; and tidying and vacuum cleaning the living room.

### Microorganisms for inoculation of gypsum boards (parts II and III).

For sampling of settling dust, EDCs were used, each EDC having a surface exposure area of 0.0209 m^2^ (19 by 11 cm). EDC samplers were used because the concentration of airborne inhalable fungi correlates significantly with the number of fungi sampled using EDC samplers in homes, and because EDC samplers can be used for long-term sampling of airborne fungi (3). The EDCs were placed at heights between 1.25 and 1.80 m above floor level. Ten EDCs were placed in two neighboring offices (ground floor, 2 occupants in each) (sample A) and in the living rooms of two homes (2 and 4 occupants) (samples B and C). In another home (4 occupants, one family house, ground floor), 32 EDCs were placed in the living room (sample D). Airborne dust settled for 26 days to allow for a high fungal diversity. The samplings were carried out in March 2012 (sample A), in September to December 2011 (samples B and C), and in November 2012 (sample D). Dust from each EDC was extracted in 20 ml pyrogen-free water with 0.05% Tween 20 by orbital shaking (300 rpm, 60 min, at room temperature [RT]). The particle suspensions were harvested from the cloths. Sample A consisted of a suspension of dust from 18 cloth, sample B from 15 cloth, sample C from 15 cloth, and sample D from 16 cloth. The suspensions were immediately used for inoculation of gypsum boards, while a subsample from each dust suspension was used for measuring the concentrations of microorganisms.

### Inoculation and incubation of gypsum boards (parts II and III).

The concentrations of fungi and bacteria in the inoculum for the gypsum boards (parts II and III) were as follows: sample A, 6.3 CFU fungi/ml and 22 CFU bacteria/ml; sample B, 23 CFU fungi/ml and 13 CFU bacteria/ml; sample C, 29 CFU fungi/ml and 19 CFU bacteria/ml; and sample D, 80 CFU fungi/ml and 7.0 CFU bacteria/ml. Gypsum boards (Knauf, Danogips, Denmark) (14 by 14 cm; mean weight, 178 g) were sterilized by heating at 120°C for 3 h. Then, the boards were soaked in distilled Milli-Q water until saturation (105 ml for 2 h) (19). The suspensions of microorganisms were applied to the wet gypsum boards (5 ml per board) and spread using a Drigalski spatula. Next, the gypsum boards were placed onto racks in autoclaved stainless steel boxes with tightly fitting glass covers (8) and covered with a white cotton fabric. Sterilized, saturated K_2_SO_4_ suspensions were used to regulate the RH in the boxes (36) to keep an RH of 97%. The fungal colonization of the gypsum boards was observed every third day; after 32 days, most of the surface of the gypsum boards was colonized, and the growth did not appear to develop further. After 40 days of incubation at RT, the RH was lowered in one-half of the boxes by applying 666 g of silica gel per gypsum board. Incubation then continued for another 10 days. This setup simulates two scenarios: one in which fungus-infested building materials are wet, e.g., due to flooding, and another one in which fungus-infested building materials dry out after a flooding (20). Throughout this paper, those gypsum boards that were incubated at high RH for the full incubation period will be referred to as “wet,” while gypsum boards incubated at low RH for the last 10 days of the incubation period will be referred to as “dry.”

### Aerosolization of fungal particles by the P-FLEC.

The particle-field and laboratory emission cell (P-FLEC; Chematec, Denmark) was used for aerosolizing of fungal particles (19). The P-FLEC was placed on the fungal cultures, and an airflow was directed toward the surface at an angle of 45°. The jets were scanned over the surface area of a 130-cm^2^ gypsum board. A bar with 10 0.8-mm nozzles was rotated in a circular movement 1.0 cm over the surface; one complete rotation was adjusted to last 60 or 120 s. The P-FLEC ran for 3 min or two or three times 3 min on the same board but was moved 4 mm between each period. Two different flows were used, 5.0 and 10 liters/min, which resulted in mean velocities over the surface of 1.5 m/s and 3.0 m/s, respectively. The aerosolized particles were transported by the airflow to the outlet at the top and were sampled using Gesamtstaub-probenahme (GSP; CIS by BGI, Inc., Waltham, MA, USA) inhalable samplers or measured by an aerodynamic particle sizer (APS; catalog no. 3320; TSI Inc., USA) that samples 51 size ranges between 0.54 and 19.8 μm, or they were (in part III) transported to the mouse exposure chamber. The RH of the air in the P-FLEC was measured by a humidity and temperature probe (HM141; Vaisala, Finland).

In part III, two P-FLECs were used at the same time, and they were started 1 min apart. The P-FLECs ran for 120 s, were moved 4 mm, and then ran for another 120 s on the same board, after which the gypsum board was replaced by a new gypsum board. To try to reach the same exposure level from dry and wet gypsum boards, two flows were used: 5.0 liters/min for dry gypsum boards and 10 liters/min for wet gypsum boards. The first experiment ran for 1 h on gypsum boards inoculated with fungi in home dust (inoculum sample D). The experiment was repeated with fungi in dust from a home (sample C) and offices (sample A) as inoculum. The repeated experiments ran for 50 min. As a reference, particles were generated for 15 min from a gypsum board without fungal colonization.

### Particle sampling in exposure chamber (part III).

**FIG 1:**
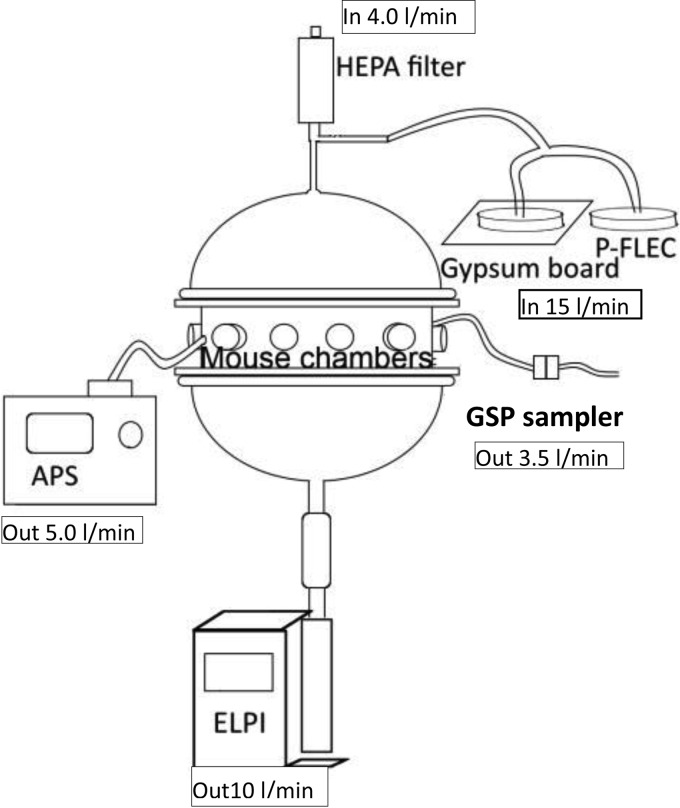
Schematic illustration of the generation and exposure system. For explanations of abbreviations, see Table 2.

The measurement setup used with the mouse exposure chamber is illustrated in Fig. 1. The exposure chamber is a vertical stainless steel cylinder (diameter, 40 cm; height, 37 cm; volume, 0.024 m^3^) with a hemispherical lid and a bottom made of glass. An amount of 15 liters of air with fungal spores/min was transported into the mouse exposure chamber by the P-FLEC, and another 4 liters clean air/min was transported into the chamber. Particles in the exposure chamber were measured online with 1-s time resolution with an APS (flow rate, 5.0 liters/min). The APS was mounted adjacent to the mice's noses. Concentrations of particles are expressed as follows: number (twa)/m^3^ air, where twa is the time-weighted average. In addition, aerodynamic particle size distribution was measured with an electrical low-pressure impactor (ELPI+; Dekati Ltd., Tampere, Finland). This device has 15 stages and collects particles in 14 size channels between 6 nm and 10 μm with 1-s intervals and samples at a flow rate of 10 liters/min. Spores on the ELPI collection plates were extracted and quantified as described. Particles were also collected on polycarbonate filters (pore size, 0.8 μm; flow, 3.5 liters/min) using a GSP sampler. The sampling was performed twice for 1 h of aerosolization and was repeated with sampling times of 50 min.

Particles were also sampled using a micro-inertial impactor with three sampling stages for single-particle analysis in scanning electron microscopy (37). The three impactor stages efficiently collect particles from a d_ae_ of ∼50 nm to 3 μm; the cutoff between the small and the medium stages is around 800 nm, and that between the medium and the large stages is around a d_ae_ of 2 μm. There is an overlap in sizes between the medium and the large stages, both containing particles with a geometric diameter of around 2.5 μm. Particles were collected on nickel plate (large stage) and nickel transmission electronic microscopy (TEM) grids with Formvar carbon foil (Plano, Wetzlar, Germany) (small and medium stages).

### Extraction of fungi and bacteria for cultivation.

The dust on the sampling filters for quantification of microorganisms was extracted in sterile 0.05% Tween 80 and 0.85% NaCl aqueous solution by shaking for a 15-min period (500 rpm) at room temperature. DGI filters were extracted in 4.0 ml, GSP filters in 6.0 ml, and ELPI filters in 5.0 ml.

### Quantification of fungi and bacteria by cultivation.

Plate dilutions were performed directly after extraction of the dust from EDCs, the polycarbonate filters from the DGI, ELPI, and GSP samplers. The number of fungi cultivable on dichloran glycerol agar (DG 18 agar; Merck, Germany) at 25°C was determined after 3, 7, and 14 days of incubation. Mesophilic bacteria from the ELPI were quantified after 3 and 7 days of incubation on 100% nutrient agar (Oxoid, Basingstoke, United Kingdom) with actidione (cycloheximide; 50 mg/liter; Serva, Germany). Fungi in the homes (part I) and the exposure chamber (part III) are expressed as CFU per cubic meter of air (twa).

### Fluorescence microscopy.

To see if fungal particles were present on the ELPI collection plates for particles smaller than spore size, 1 ml of the suspensions from stages 7, 9, 13, and 15 were filtered through a polycarbonate filter (0.4 μm; Nuclepore, Cambridge, MA, USA) and stained in 20 ppm acridine orange (Merck Millipore, Darmstadt, Germany) in acetate buffer for 30 s. Fungal particles were identified at a magnification of ×1,250 using epifluorescence microscopy (Orthoplan; Leitz Wetzlar).

### Identification of airborne fungi using MALDI-TOF.

Fungi sampled in home I (part I) and in the exposure chamber (part III) were identified using MALDI-TOF. The MALDI-TOF analysis was performed using the MALDI-TOF MS Biotyper System (Bruker Daltonics, Bremen, Germany). Fungi were grown overnight in Sabouraud growth medium (SGM; Oxoid, Hampshire, England). The growth medium was washed out twice from the samples. A small amount of fungal hyphae was suspended in a 70% ethanol solution and centrifuged at 13,000 × *g* for 2 min. The pellet was left to dry for 5 min at RT. Formic acid was added to the sample, the pellet was resuspended, and an equal volume of acetonitrile was added to the suspension. The sample was centrifuged again at 13,000 × *g* for 2 min, and 1 μl of the supernatant was spotted onto the ground steel target plate (Bruker Daltonics, Bremen, Germany). The droplet was dried at RT, overlaid by 1 μl of matrix (Bruker Daltonics, Bremen, Germany), and then fungi were identified. A Microflex LT mass spectrometer (Bruker Daltonics) was used for the analysis, and spectra were analyzed using Bruker Biotyper 3.1 software with the BDAL standard library and filamentous library 1.0.

### Quantification of endotoxin and β-glucan.

Endotoxin and β-glucan were measured in the GSP samples from the exposure chamber (*n* = 7). Endotoxin was measured because it is a strong inflammagen that is always present in normal indoor air. Samples used for endotoxin quantification were centrifuged (1,000 × *g*) for 15 min. The supernatant was analyzed in duplicate for endotoxin using a chromogenic kinetic Limulus amoebocyte lysate test (LAL; Kinetic-QCL endotoxin kit; Lonza, Walkersville, MD, USA). A standard curve obtained from an Escherichia coli O55:B5 reference endotoxin was used to determine the concentration in terms of endotoxin units (EU) (10.0 EU ≈ 1.0 ng). Extracts from 7 GSP-polycarbonate filters were analyzed in duplicate for β-glucan using the kinetic, chromatic Fungitic G test (Seikaga Co., Tokyo, Japan). The triple-helix structure of the β-glucan was made water soluble by adding 0.3 M NaOH and incubating for 60 min. The data are presented in nanograms per cubic meter of air.

### Environmental SEM.

Samples were first investigated untreated. Some samples were then plasma coated with a thin (maximum, 3-nm) layer of gold to achieve high-quality images. Environmental scanning electron microscopy (ESEM; Quanta 200 FEG; FEI, Eindhoven, The Netherlands) was performed in low vacuum mode at 0.3-mbar chamber pressure. The instrument was operated with an electron beam acceleration voltage of 10 kV at spot size 3 for imaging of secondary electron images (Everhard Thornly detector [ETD]) and at 20 kV at spot size 4 for energy-dispersive X-ray (EDX) analysis; the latter was performed in high vacuum mode. No change in morphology of the particles was observed after pressure change. For high-resolution secondary electron images, a Helios focused ion beam (FIB)/ESEM was used on the coated samples. No systematic difference in the size and morphology of particles was observed between the two sets of images, and both were thus considered valid for image analysis.

Image analysis was performed using the particle size analyzer macro (PSA_r12; GNU General Public License v3) for the open-source software ImageJ (W. Rasband), where the average projected area diameter (also called area-equivalent diameter) of single particles was calculated from the number of pixels per area.

### PCR and Sanger sequencing.

One fungal isolate that was not identified using MALDI-TOF was sent for Sanger sequencing at Beckman Coulter Genomics (Takeley, Essex, United Kingdom). Isolation of total DNA was performed using the PowerSoil DNA isolation kit (MoBio, Copenhagen Biotech Supply, Copenhagen, DK) as described previously (15).

### Treatment of data.

In part II, the spore release potentials as a function of time and different places on the gypsum boards were compared using a paired *t* test. Amounts of fungal particles aerosolized from wet and dry gypsum boards as a function of two airflows and ratios of first to second place were compared using general linear models in SAS 9.3. In parts II and III, the geometric mean (GM) diameter (d_g_) of aerosolized particles was calculated from the APS data as follows: *d_g_* = exp (Σ*n_i_* log *d_i_*/*N*), where *n_i_* is the measured number of particles, *d_i_* is the geometric midpoint of the interval, and *N* is the total number of particles; furthermore, the geometric standard deviation, σ_*g*_, was calculated as follows: log σ_*g*_ = [Σ*n_i_* (log *d_i_* − log *d_g_*)^2^/(*N* − 1)]^1/2^ (34). In part III, concentrations of aerosolized fungi (in log CFU per cubic meter) and β-glucan (in log nanograms per cubic meter) from wet and dry gypsum boards and in the repeats were compared using analysis of variance (ANOVA) in SAS 9.3.

## RESULTS

### Part I: airborne fungal species in Danish homes.

In the airborne dust sampled from 27 homes, the three dominating species in each home in terms of concentration covered 22 different fungal species. Penicillium glabrum was among the three most abundant fungal species in 13 of 27 homes, while Penicillium brevicompactum and Penicillium camemberti were among the three most abundant fungal species in 10 of 27 homes (Table 3). Fungal species associated with water damage, according to the environmental relative moldiness index (ERMI) (38), are labeled group 1 species and are indicated in Table 3 with a superscript *d*; fungal species not associated with water damage (group 2) according to the ERMI are indicated with a superscript *e*. Eleven fungal species belong to group 1 species.

**TABLE 3:** Dominating fungal species found in airborne settled dust samples from 27 homes and fungal species found in airborne dust from home I and in aerosols in the mouse exposure chamber^*a*^

Genus and species	No. of homes (part I) where the species is among the three most frequent found fungal species (in terms of concn in 27 homes)	Present in home (part I)^*b*^	Present in aerosol in exposure chamber (part III) from GB inoculated with fungi^*c*^ found in:	
			Home dust	Office dust
Aspergillus				
A. glaucus	0	+	−	−
A. nidulans	2	+	−	+
A. niger^*d*^	3	−	+	−
A. ustus^*e*^	2	−	+	+
A. versicolor^*d*^	7	+	+	+
Eurotium amstelodami^*d*^	2	−	−	−
Botrytis cinerea	2	+	−	−
Chaetomium globosum^*d*^	2	−	−	+
Cladosporium herbarum^*e*^	4	+	+	+
Fusarium proliferatum	3	−	−	−
Penicillium				
P. brevicompactum^*d*^	10	+	+	+
P. camemberti^*d*^	10	+	+	−
P. chrysogenum^*e*^	4	+	+	+
P. commune^*d*^	3	+	+	+
P. copticola	1	−	−	−
P. corylophilium^*d*^	1	−	−	−
P. cigitatum	4	−	−	−
P. expansum	0	+	−	−
P. glabrum^*d*^	13	+	+	+
P. olsonii	2	+	−	+
P. purpurogenum^*d*^	1	−	−	−
P. rugulosum	1	−	+	−
Stachybotrys chartarum^*d*^	1	−	+	−
Wallemia sebi^*d*^	4	+	−	+

a Symbols and abbreviations: +, present; −, absent; GB, gypsum board.

b Species identified in aerosols sampled during 1 h in different rooms in home I (part I) (see Fig. 2).

c Fungal species in aerosols generated from fungi growing on gypsum boards; gypsum boards were inoculated with fungi in dust from a home (sample D) or from offices (sample A) (part III).

d Group 1 fungi commonly present in U.S. homes and associated with water damage according to ERMI.

e Group 2 fungi commonly present in U.S. homes but not associated with water damage according to ERMI (87).

We attempted to identify all cultivable fungal species sampled in home I during 1 h and found that most belonged to the genus Penicillium, which covered 7 species. Thirteen different fungal species were found (Table 3). A person moving through the different rooms in home I would thus be exposed to at least 13 different species and 5 different genera in different concentrations. Some isolates were not identified, and therefore the total sum of species, in some rooms, was less than 100% (Fig. 2).

**FIG 2:**
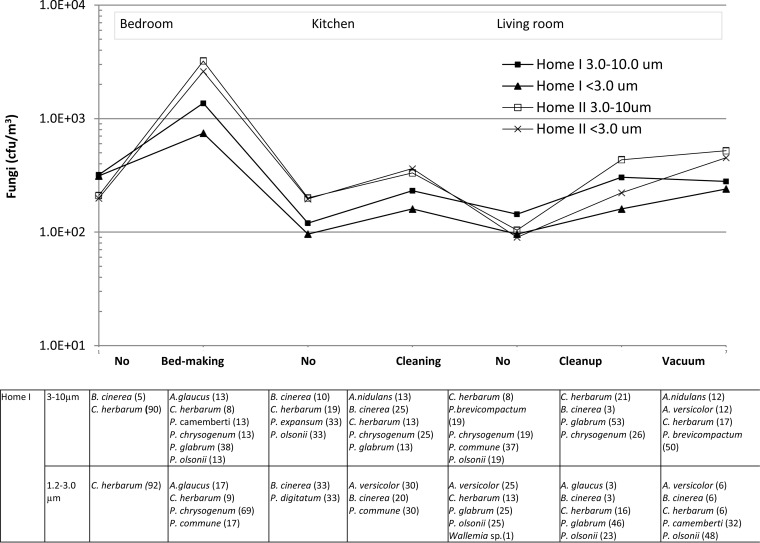
Variation in exposure (in CFU per cubic meter of air) to airborne fungi (size fraction, 1.2 to 3.0 μm or 3.0 to 10.0 μm) in rooms with different activities; measurements were performed in two homes. Fungal species in the two size fractions are identified for home I samples, the numbers in parentheses are the percentages of each species. “No” indicates measurements taken when a person is present in the room but no activity occurs.

### Variation in concentration of fungi in air.

Exposure of a passive person mimicked by a stationary sampler in a home varies with places and activities in the home (Fig. 2). The exposure level in home I varied by a factor of 10; in home II, the exposure level varied by a factor of 30. The highest exposure was found during bed making. The concentration of some fungal species increased during activities. Cladosporium herbarum was always present in the air, sometimes being the dominating species, while sometimes other fungi such as Botrytis cinerea, P. brevicompactum, P. glabrum, or Penicillium chrysogenum dominated. The different fungal species were found in both size fractions, but C. herbarum tended to be found in higher concentrations in the 3.0- to 10-μm fraction while Aspergillus tended to be found in higher concentrations in the 1.2- to 3.0-μm fraction (Fig. 2).

### Part II: amounts of fungal particles aerosolized from gypsum boards.

The amounts of fungal spores aerosolized from gypsum boards during 3 min of exposure to an airflow of either 1.5 m/s or 3.0 m/s were measured as the numbers of spores and as the calculated weight of spores aerosolized from a 130-cm^2^ surface of gypsum board. The largest amounts were released when the fungus-infested gypsum boards were dried before air exposure, but a twice as high airflow resulted in similar amounts from wet gypsum boards (Table 4).

**TABLE 4:** Amounts of fungal spores aerosolized per min during 3 min of exposure of fungus-infested gypsum boards and geometric mean diameters of aerosolized particles

Inoculum	Culture humidity level (RH%)	*n*	Airflow (m/s)	Calculated wt (μg/130 cm^2^/min)^*a*^^,^^*b*^ for:		Inhalable no. of fungi (CFU/130 cm^2^/min)^*a*^	Geometric mean diam (μm)^*c*^
				PM_20_	PM_2.1_		
Sample A fungi in office dust	Wet (57)	6	1.5	11.3, **12.1** (2.5–16.8) b	0.11, **0.13** (0.021–0.27) b	3.51 × 10^5^, **3.18** × **10^5^** (1.26 × 10^4^–9.27 × 10^5^) b	2.79 (2.72–2.88) σ_g_ = 1.01–1.32
	Dry (26)	6	1.5	41.6, **43.5** (12.7–86.5) a	0.21, **0.24** (0.10–34) b	4.61 × 10^6^, **2.80** × **10^6^** (1.45 × 10^5^–1.60 × 10^7^) a	2.91 (2.92–2.96) σ_g_ = 1.15–1.26
Sample B fungi in home dust	Wet (62)	12	1.5	10.2, **5.4** (2.4–34) b	0.66, **0.82** (0.078–1.46) b	7.23 × 10^5^, **5.77** × **10^5^** (2.76 × 10^4^–2.63 × 10^6^) b	2.20 (1.8–2.5) σ_g_ = 1.17–1.23
	Dry (27)	12	1.5	33.6, **31.8** (18.0–86) a	7.8, **6.8** (2.0–15.4) a	6.98 × 10^6^, **4.82** × **10^6^** (1.05 × 10^6^–1.74 × 10^7^) a	2.09 (1.92–2.41) σ_g_ = 1.20–1.52
Sample C fungi in home dust	Wet (64)	6	3.0	31.4, **24.9** (11.0–63.1) a	1.27, **1.05** (1.23–15) a	3.11 × 10^6^, **1.05** × **10^6^** (1.33 × 10^5^–9.02 × 10^6^) a	2.18 (1.90–2.49) σ_g_ = 1.17–1.50
	Dry (23)	6	1.5	38.0, **34.7** (11.7–77.4) a	1.71, **1.27** (0.92–19) a	4.92 × 10^6^, **3.92** × **10^6^** (2.50 × 10^5^–1.00 × 10^7^) a	2.27 (2.02–2.51) σ_g_ = 1.15–1.39

a Values are averages, medians (in boldface), and ranges (in parentheses). Values in the same column followed by the same lowercase letter are not statistically significantly different. PM_2.1_, particulate matter with an aerodynamic diameter below 2.1 μm; PM_20_, particulate matter with anaerodynamic diameter below 20 μm.

b The mass is calculated from APS data with the assumption that particles are spherical and have a density of 1 (88).

c σ_g_, geometric standard deviation.

### Spore release potential.

**FIG 3:**
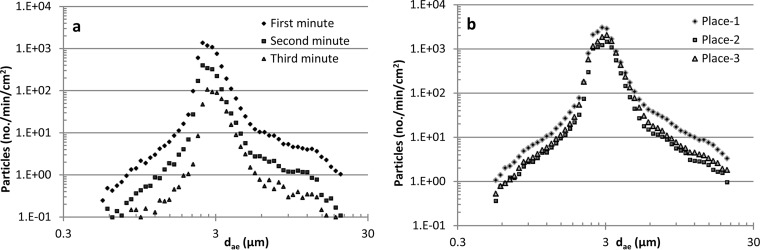
Example of the number and size distribution of particles aerosolized from fungus-infested gypsum boards (inoculum sample A) as affected by an air exposure (1.5 m/s) in the first, second, and third minutes of exposure of the same area (RH = 54%) (a) and in the first minute (of 3 min) in three different places on the same board (RH = 23%) (b).

The spore release potential of fungus-infested gypsum boards decreased by time of air exposure (Fig. 3a). Thus, the sums of particles aerosolized from wet and dry gypsum boards, respectively, in the first minute were on average 3.1 (GM = 2.9, *n* = 18, *P* < 0.001) and 2.9 (geometric mean [GM] = 2.9, *n* = 17, *P* = 0.01) times higher than during the second minute; the same ratios for first and third minutes were 7.6 (GM = 7.2, *n* = 18, *P* < 0.001) and 7.9 (GM = 7.6, *n* = 17, *P* < 0.001) times.

When the P-FLEC was moved 4 mm on the same board between 3-min samplings, the exposure also decreased; the sum of particles aerosolized from wet and dry gypsum boards, respectively, in the first place was on average 3.8 (GM = 3.7, *n* = 6, *P* = 0.0065) and 5.6 (GM = 5.3, *n* = 5, *P* = 0.012) times higher than on the second place; the same ratios for first versus third place were 3.5 (GM = 3.1, *P* = 0.018) and 7.3 (GM = 6.4, *P* = 0.012) (an example is shown in Fig. 3b). The ratio of first to second place tended to be higher for dry than for wet gypsum boards (*P* = 0.076). The ratio of first to third place was higher for dry than for wet gypsum boards (*P* = 0.049).

### Size distribution of aerosolized fungal particles.

**FIG 4:**
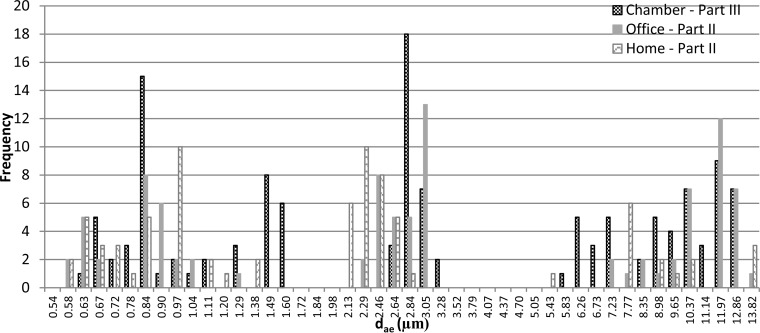
Frequency of modes of particles (APS data) with different aerodynamic diameters (d_ae_) in aerosols from each gypsum board (inoculum samples A and B, *n* = 30, part II), and every second minute in the exposure chamber (part III, wet gypsum board, inoculum sample D).

The particle size distribution showed the largest mode for particles with a d_ae_ of about 2 to 3 μm (examples are shown in Fig. 3a and b). With office (sample A) and home (sample B) dust as inoculum, 13 of 30 and 10 of 30 aerosols had modes at 3.05 μm and 2.29 μm, respectively (Fig. 4). Modes with smaller and larger particles were also seen, but with lower concentrations than those with the mode around 2 to 3 μm. The average geometric mean diameters (d_g_) were between 2 and 3 μm (Table 4).

### Part III: concentrations in mouse exposure chamber.

**FIG 5:**
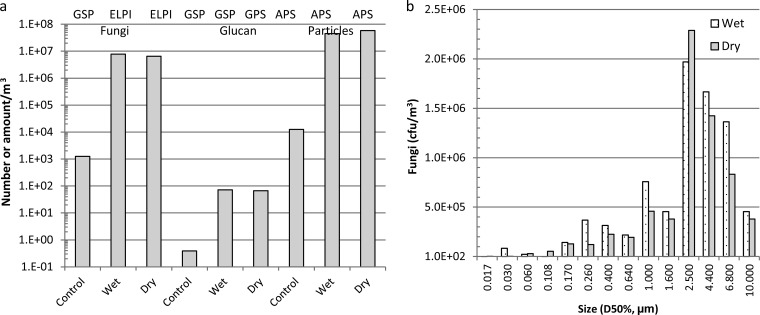
Measurement of fungi in CFU (a and b), β-glucan in nanograms (a), and particles between 0.54 and 19.8 μm (a) in a mouse exposure chamber. Fungi were aerosolized from wet and dry gypsum boards (inoculum sample D); fungi were sampled by an ELPI in 14 size fractions (b); the 14 size fractions are pooled in panel a; fungi were sampled by a GSP sampler (a); β-glucan was sampled by a GSP sampler (a); the number of particles was determined by an APS (a). Time-weighted average concentrations were measured during a 1-hour exposure study and a control study (2 times 15 min). For abbreviations, see Table 2.

The concentrations of airborne fungi and particles were measured with a GSP sampler, ELPI, and APS, and examples (inoculum: sample D) are presented in Fig. 5a and b. It was possible to reach the same level of fungi from wet and dry gypsum boards by using two different airflows. Thus, no significant difference between fungi aerosolized from dry and wet gypsum boards was found when fungi were measured as CFU (*P* = 0.52) and as β-glucan (*P* = 0.62). The concentrations of aerosolized fungi from wet versus dry gypsum boards were as follows: 1.4 × 10^7^ CFU/m^3^ versus 9.3 × 10^6^ CFU/m^3^ and 74 ng glucan/m^3^ versus 69 ng glucan/m^3^ (inoculum sample A); 9.9 × 10^6^ CFU/m^3^ versus 1.1 × 10^7^ CFU/m^3^ and 70 ng glucan/m^3^ and 74 ng glucan/m^3^ (inoculum sample C); 7.8 × 10^6^ CFU/m^3^ versus 6.5 × 10^6^ CFU/m^3^ and 72 ng glucan/m^3^ versus 67 ng glucan/m^3^ (inoculum sample D). No differences were found between the experiments (for CFU, *P* = 0.16; for β-glucan, *P* = 0.31); when comparing the concentrations (CFU) in each size fraction in the ELPI, no significant differences were found between the experiments (all *P* values > 0.05).

**FIG 6:**
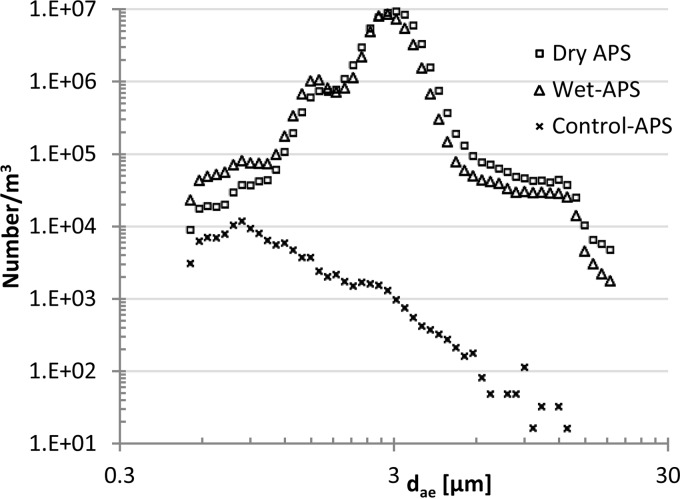
Time-weighted average concentration (number per cubic meter) of fungal particles as a function of aerodynamic diameter (d_ae_, μm) in a mouse exposure chamber as measured by an APS during a 60-min period. Aerosols were generated using two P-FLECs from fungi grown on gypsum boards (inoculum sample D) that were wet or dry or from a gypsum board without fungal growth (control).

In all experiments, most fungal spores were present in the 2.5-μm fraction (Fig. 5b). The d_g_ for particles in the aerosol chamber for wet was 2.69 μm (σ_g_ = 1.35), for dry 2.87 μm (σ_g_ = 1.31), and for control 0.98 μm (σ_g_ = 1.08). The average number and size distribution of particles in the aerosol chamber during the 60 min of aerosolization from fungus-infested dry and wet and control gypsum boards are shown in Fig. 6. Cultivation and microscopy showed that a few spores were also present in the ELPI level for particles expected to be smaller than spore size (Fig. 5b).

**FIG 7:**
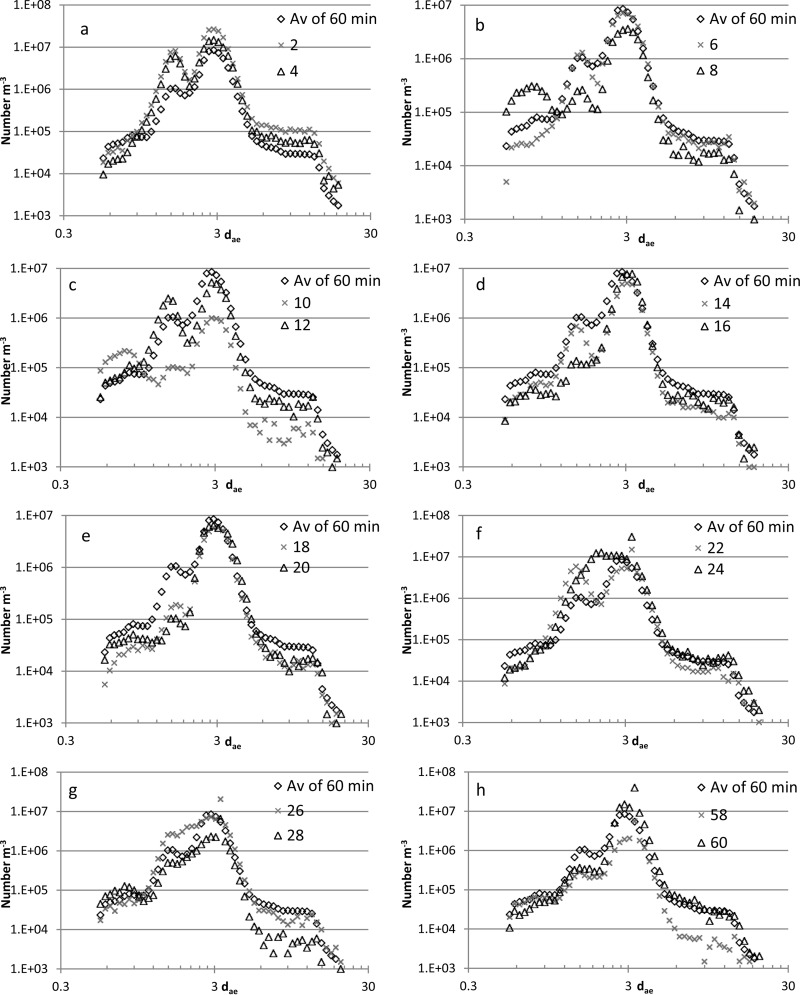
Average concentrations (number per cubic meter) of fungal particles as a function of aerodynamic diameter (d_ae_, in micrometers) in a mice exposure chamber as measured by an APS during a 60-min period (a to h) and averages of 2-min intervals for the first 28 min (a to g) and for the last minutes (minutes 57 and 58 and minutes 59 and 60) (h). Aerosols were generated using two P-FLECs from mixed fungal species grown on gypsum boards. Examples are given for wet gypsum boards with sample D as inoculum.

Averages of 2-min periods compared to the average of the whole period of 60 min are shown in Fig. 7 (example with boards inoculated with sample D). In Fig. 4, frequencies of modes of 2-min averages are shown. Most of the fungal spores were present in the fraction with particles of around 2 to 3 μm, but modes of smaller and larger particles were also observed. The maximum variations in concentration (2-min averages) of particles between 2.6 and 3.1 μm during the 60 min of aerosol generation were of a factor of 27 and 23, respectively, for aerosolization from cultures on wet and dry gypsum boards. The size distribution of particles over time reveals different particle sizes (Fig. 7).

The concentrations of airborne endotoxin and bacteria in controls (no-spore aerosolization) were 0.9 EU/m^3^ and 208 CFU bacteria/m^3^. The average concentrations (ranges) of airborne endotoxin and bacteria from infested wet gypsum boards were 1.2 EU/m^3^ (0.8 to 1.4 EU/m^3^) and 236 CFU bacteria/m^3^ (152 to 339 CFU/m^3^), and for dry gypsum boards they were 1.3 EU/m^3^ (1 to 1.4 EU/m^3^) and 192 CFU/m^3^ (129 to 294 CFU/m^3^).

### Environmental scanning electron microscopy.

**FIG 8:**
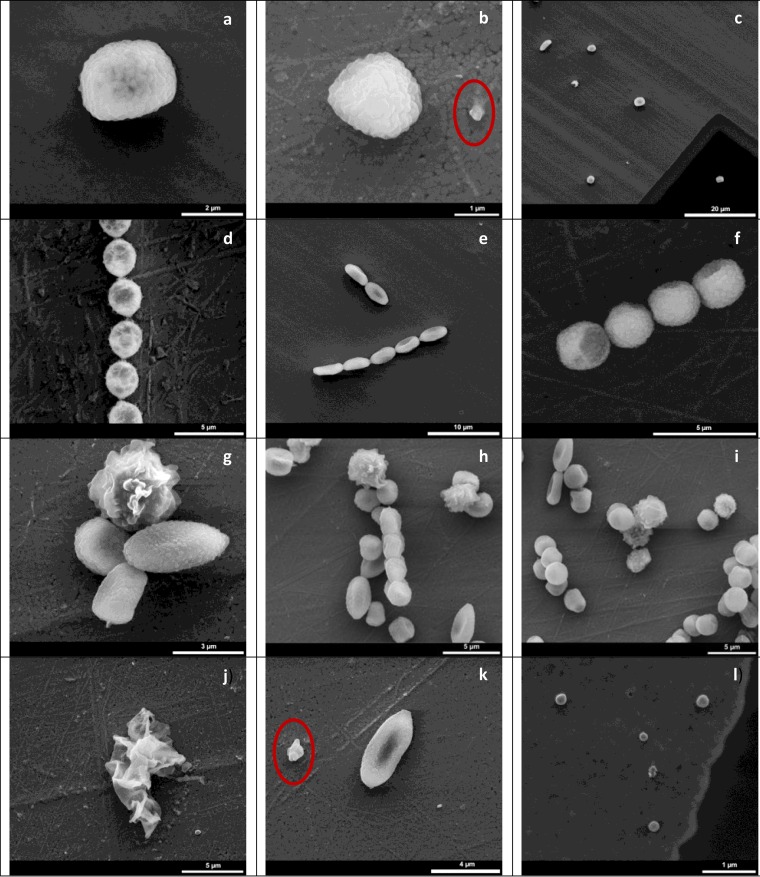
Fungal particles sampled from aerosols in a mouse exposure chamber. Aerosols were generated from fungus-infested gypsum boards which were wet or dry. Fungi were present as single spores (a, b, c, and k) of different sizes and shapes, chains of 3 spores from one species (d, e, f), clusters of spores from different species (g, h, i), residues (j; also indicated by circles in panels b and k), and small instable residues (l).

Fungal spores were found in the air in the mouse exposure chamber mainly as single spores (Fig. 8a, b, c, and k) of different sizes and shapes, but also as chains of spores from one species (Fig. 8d, e, and f), as clusters from different species (Fig. 8g, h, and i), and as fragments (Fig. 8b, j, and k). The average projected area diameter for single spores ranged from 1.7 to 3.2 μm (average, 2.3 μm). Taking into account the clusters and fungal residues, the wider-size distribution ranges from 1.2 to 9.6 μm with an average diameter of 3.5 μm. On the stage with particles smaller than 800 nm, spherical particles with an average diameter of 0.19 μm were observed (examples are shown in Fig. 8l). These particles resisted the applied pressure conditions but were instable to the electron beam.

One microscope view was studied further: 766 spores were present, consisting of four different spore types; 10 chains of the spore type seen in Fig. 8f contained from 3 to 16 spores; 3 chains of the spore type seen in Fig. 8e contained 3 to 11 spores; the final two types of spores were found as single spores or as clustered with other species.

### Fungal species.

Identification of fungi showed the presence of 15 species in aerosols from fungus-infested gypsum boards: 11 different species were found in aerosols generated from gypsum boards inoculated with fungi in dust both from a home and from offices. Six different Penicillium species and four different Aspergillus species were found. All species aerosolized from the fungus-infested gypsum boards were among the dominating species found in 27 homes, and 7 of these species were also found in home I (Table 3).

## DISCUSSION

Aerosols with 11 different fungal species were generated directly from fungus-infested gypsum boards and into a mouse exposure chamber by exposing the boards to an airflow. Thus, it is possible through this system to expose mice to aerosols containing a mixture of fungal species. The species present in the aerosols correspond well with species present in airborne dust in 28 Danish homes (Table 3).The maximum mode of the airborne fungi in the mouse exposure chamber was most frequently 2.8 μm (Fig. 4), and a maximum mode of this size was also seen in aerosols measured directly in the P-FLEC (Fig. 3). The average concentration of fungi in the exposure chamber corresponds with the level measured during remediation of a moldy building (5). The β-glucan concentration was approximately 25 times higher than exposures found in suburban homes (39). The variation in exposure between 2-min averages was up to a factor of 27, and this seems realistic in view of the variation found in home I and home II.

The APS profiles showed that particles of different sizes were aerosolized over time, reflecting the dominance in terms of concentrations of different species. Most spores were present in the air as single spores (Fig. 5b, 6, 7, and 8c), but clusters of different species (Fig. 8g, h, and i) and chains of spores (Fig. 8d, e, and f) were also seen. The chains of spores may have detached directly from a conidiophore. The clusters and chains were reflected in the APS data with modes for d_ae_ of 6 to 12 μm. At the home level, spores were present both as single spores and as clusters or associated with other particles since fungi were present both in the 1.2- to 3.0-μm and the 3.0- to 10-μm fraction (Fig. 2). The presence of conidia in chains may affect the spore inhalation and deposition. About 0.4% and 30% of particles of 12 μm and 5 μm, respectively, are respired by humans (34). In a water suspension, chains of spores may break apart into single spores, which may enter more deeply into the airways than chains of spores. Thus, the exposure method (water suspension versus aerosol) would indirectly affect the inhaled level. The aerosolization of fungi as single spores versus chains of spores seems in this study to be species dependent; in another study, aggregate formation of spores from the same species seems also to be species dependent (31). Therefore, the composition of fungal species entering the airways may also be affected by the exposure method.

For the generation of aerosols at the exposure chamber level, an area of 0.195 m^2^ fungus-infested gypsum board was exposed to air jets of 1.5 or 3.0 m/s for 1 h. In comparison, a destructive inspection of a gypsum wall in leaky rooms showed that 26% of the entire wall area was visibly colonized by fungi; the same value in nonleaky rooms was 1.5% (40). In a fictive room of 2.5 by 8.0 by 6.0 m (120 m^3^) with a total wall and roof area of 118 m^2^, a 26% or 1.5% fungus-infested area corresponds to 30.7 m^2^ or 1.77 m^2^ fungal coverage; this corresponds to a 157 or 9 times larger area than the area causing the measured exposure (6 to 8 × 10^6^ CFU fungi/m^3^) in the mouse exposure chamber.

The number of spores aerosolized per minute for wet gypsum boards was between 97 and 7 × 10^4^ CFU/cm^2^/min and for dry between 1 × 10^3^ and 1 × 10^5^ CFU/cm^2^/min. In a similar study, the average number of aerosolized spores was 4 × 10^3^ CFU/cm^2^/min for wet gypsum boards and 3 × 10^4^ CFU/cm^2^/min for dry gypsum boards (20). In a study with spore aerosolization from naturally infested building materials in homes, the spore aerosolization was smaller (from 10^2^ to 10^3^ CFU/cm^2^/min) (41). The spore release potential from fungally infested gypsum boards decreased over time, but the decrease could be reduced by moving the P-FLEC a few millimeters. Furthermore, the difference in spore release between gypsum boards could partly be levelled by aerosolizing particles from two gypsum boards at the same time. There was a tendency to a greater reduction in spore release after 3 min of air exposure of dry than of wet infested gypsum boards; this may be because more spores are initially aerosolized from the dry gypsum boards.

In home I, 13 different airborne fungal species were identified, and before activities, C. herbarum dominated in terms of frequency. In accordance with an earlier study (42), it tended to be found in higher concentrations in the particle fraction with the largest particles. It was also among the three dominating species in 4 of the 27 homes, and it was the most frequently found fungus in Danish homes in 1970 to 1971 (43). At the genus level, Cladosporium exposure has been associated with reduced lung function among schoolchildren (44). The fungus B. cinerea is able to grow on gypsum boards, vegetables, and fruits (8). It was found in the bedroom and kitchen and was dominating in 2 homes, it has previously been found in Danish homes (43), and people can be allergic to it (45). Seven Penicillium species were found in home I and six in the aerosols from the gypsum boards. Twelve Penicillium species were among the dominating species in the 27 homes. Five species (P. brevicompactum, P. camemberti, P. chrysogenum, P. commune, and P. glabrum) were found both in the air in the homes and in the aerosols generated from the gypsum boards. Some of these species have earlier been found in homes or offices (38, 46). We have found no publications dating later than the 1970s on airborne fungal species in Danish homes or offices. In a study in Danish homes, Penicillium, identified to the genus level, was the second most frequently found genus (43). In many exposure studies, Penicillium has been identified only to the genus level (47–50); the MALDI-TOF method allows identification of 23 Penicillium species. Epidemiological studies have shown an association between exposure to Penicillium and increased risk of wheeze, persistent cough, and higher asthma severity score (47) and between Penicillium in dust and wheeze (14). Three Aspergillus species were found in home I, four species in the aerosols from the gypsum boards, and four species were among the dominating species in the 27 homes. Two of these species have also been found in the air in Finnish homes or offices (51) and in U.S. homes (52). Aspergillus niger and Aspergillus versicolor, which were aerosolized from the gypsum boards, have also been found in surface samples from gypsum boards in homes (53). Aspergillus ustus and A. niger were found in homes and in aerosols generated from the gypsum boards; the presence of A. ustus in home dust is predictive of development of childhood asthma (13), and high concentrations of A. niger have been found in homes of asthmatic children (54). Stachybotrys chartarum, found in the aerosols from the gypsum boards, has attracted attention due to its adverse health effects (55).

This study shows that within 1 h in a home a person is exposed to different fungal species and concentrations in different rooms. This seems to be a consequence of different activities. Variation in fungal exposure between concentrations of cultivable fungi in morning, midday, and afternoon measurements has been found in two Finish homes (56). Thus, to appropriately study the health effects of exposure to indoor fungi, it appears to be relevant to expose the model animal to different fungal species simultaneously in different concentrations as done with the setup described in this study. The ERMI is based on fungi in surface dust in U.S. homes, and 36 widely distributed fungal species or groups of fungal species are grouped into fungi associated (group 1) and not associated (group 2) with water damage (38). By sampling indoor airborne fungi followed by cultivation on wet gypsum boards and aerosolization, we tried to simulate water damage in a home. Indeed, the species aerosolized from the gypsum boards included species categorized as group 1 fungi in the ERMI. Thus, 9 group 1 species were found in aerosols generated from the gypsum boards. Furthermore, 11 of the group 1 species were among the dominating species in the 27 homes. Three species belong to the group 2 fungi, and they were both found in airborne dust in homes and in the aerosols generated in the exposure chamber. Thus, similarities between species in U.S. and Danish homes and in aerosols generated from the gypsum boards are found. Use of PCR-based methods, as in the ERMI, may have shown even more similarities; however, this study with CFU and MALDI-TOF is done with airborne fungi while the ERMI is done with fungi in floor dust. The composition of fungi aerosolized from gypsum boards resembled that found in Danish homes, and all species found in these aerosols were among the dominating species found in 27 homes.

Mouse exposure studies are often done with one or two fungal species (Table 1), even though people are exposed to several species simultaneously. Significantly higher fungal diversity and occurrence of airway symptoms have been found among schoolchildren in moisture-damaged schools than among those attending schools without moisture problems (57). Further, factors associated with asthma seem to affect the profile of the microbial community in-house dust (12), and low diversity among fungi (58) and Cryptococcus (58) has been associated with risk of development of asthma. Dust that caused ill health effects in a working environment had a microbial composition different from that of reference dust (15). Furthermore, an editorial paper concluded that we need to obtain a better understanding of the composition of airborne microbial communities (59). Using the model described in this study, it will be possible to expose mice to several species simultaneously and thus further study the importance of fungal diversity, mixtures of species, and different concentrations. In addition, cocultivation of fungal species may be of importance, as other studies have shown that the composition of neighboring fungal populations may affect enzyme production (60).

Experimental data for mice show that about 58%, 8.2%, and 5.9% of particles of 1.0 μm deposit in the oral/nasal, tracheal/bronchial, and lung region, respectively (33). In this study, the largest mode was seen for particles around 2.6 to 3.1 μm, and this corresponds with the spore diameter of several of the species identified in the aerosol samples. As an example, aerosols generated from pure P. chrysogenum cultures have a mode at 2.8 μm, and spores have a diameter of 2.8 to 4.0 μm (61). Both APS and ESEM data show that particles smaller than spore size constitute a smaller number than the number of particles of spore size. This corresponds with the laminar flow (LAF) bench level for A. versicolor, P. chrysogenum, and Trichoderma harzianum growing on gypsum boards (19, 61). However, it contrasts with what has been seen for A. versicolor and for Cladosporium cladosporioides on agar media exposed to certain air velocities (30, 62). Fragmentation of fungi may be caused by aerosolization using a high air velocity (63). Some particles seem to have a low density (Fig. 8j) and may have been measured as smaller than their actual size by the APS. Data from the ELPI show that culturable fungi were also present in the stage where particles smaller than spore size were expected to be found; microscopy showed that this was due to the presence of spores, and this may be due to particle bouncing. Spore bouncing during sampling has been found earlier (64).

Aerosols were generated from moldy gypsum boards by an airflow; in homes I and II, work tasks aerosolized or resuspended fungi. The higher spore aerosolization from dry building materials than from wet is of importance in relation to remediation of damp buildings. During remediation of moldy offices, office workers have experienced more symptoms than before remediation (65). The authors suggest that this may be caused by an increased exposure during remediation or that recovery from damp-building-related respiratory illness is incomplete and delayed. Drying out is a normal step in managing flood damage (66), and higher concentrations of fungi have been reported during repair processes (5, 67). In epidemiological studies, indoor dampness is shown to be associated with respiratory symptoms, but a causal relationship is not well documented (1). This might partly be because other changes also occur as a consequence of dampness, such as an increased number of dust mites (68), or bacteria, and endotoxin. With the setup described in this study, it will be possible to exclude factors such as allergens from dust mites and dogs and microorganisms from other sources such as human skin or vegetables. Thus, it would be possible to study the potential health effects of fungal growth on building materials without confounding interference from other components. It is also possible to study the importance of humidity in a building material with regard to fungal exposure and associated health outcomes. In this study, the exposures to bacteria and endotoxin at the exposure chamber level were not elevated in comparison with normal indoor levels (69, 70).

As described above, the exposure to fungal aerosols generated directly from the source instead of a water suspension has several advantages. However, one disadvantage may be that it can be difficult to reproduce with exactly the same concentrations and species composition; by reproducing the study, we were able to obtain exposures to fungi at the same levels, and 7 of 11 species overlapped between two studies. This shows that it is possible to repeat an exposure level. Based on the data in Fig. 3, reuse of the same fungus-infested gypsum boards within a short period of time may be a way to repeatedly obtain more similar fungal aerosols. Another disadvantage is that the dust sampling, cultivation, and exposure method is time-consuming.

In conclusion, using the described system with aerosolization of fungi from the indoor environment growing on gypsum boards, it is possible to generate aerosols realistic in terms of composition at the species level, concentration, and particle sizes. The aerosols generated contained around 10^7^ CFU of fungi/m^3^ air. The aerosols contained 15 different fungal species, and these species were among the 24 dominating species found in 27 Danish homes. High similarities between species in aerosols generated from the gypsum boards, in Danish homes, and in U.S. homes were found. ELPI, APS, and ESEM data show that most airborne fungal spores in the exposure chamber were present as single spores, but some were also present in clusters or chains. Using mice as model animals, the system can be used to study responses to exposure to indoor fungi, e.g., associated with water damage, or to study the importance of fungal species or diversity.
